# Healthy Beyond Pregnancy, a Web-Based Intervention to Improve Adherence to Postpartum Care: Randomized Controlled Feasibility Trial

**DOI:** 10.2196/humanfactors.7964

**Published:** 2017-10-10

**Authors:** Katherine Park Himes, Heidi Donovan, Stephanie Wang, Carrie Weaver, Jillian Rae Grove, Francesca Lucia Facco

**Affiliations:** ^1^ Magee-Womens Research Institute Department of Obstetrics, Gynecology, and Reproductive Sciences University of Pittsburgh Pittsburgh, PA United States; ^2^ School of Nursing Health and Community Systems University of Pittsburgh Pittsburgh, PA United States; ^3^ Department of Economics University of Pittsburgh Pittsburgh, PA United States; ^4^ University of Pittsburgh Medical Center Department of Obstetrics, Gynecology and Reproductive Sciences Pittsburgh, PA United States

**Keywords:** postpartum visit, behavioral economics, Medicaid, intervention, usability

## Abstract

**Background:**

During the postpartum visit, health care providers address issues with short- and long-term implications for maternal and child health. Women with Medicaid insurance are less likely to return for a postpartum visit compared with women with private insurance. Behavioral economics acknowledges that people do not make exclusively rational choices, rather immediate gratification, cognitive and psychological biases, and social norms influence decision making. Drawing on insights from decision science, behavioral economists have examined how these biases can be modulated through carefully designed interventions. We have developed a Web-based tool, Healthy Beyond Pregnancy, that incorporates empirically derived concepts of behavioral economics to improve adherence rates to the postpartum visit.

**Objectives:**

The primary objectives of this pilot study were to (1) refine and assess the usability of Healthy Beyond Pregnancy and (2) assess the feasibility of a randomized controlled trial (RCT) of the intervention.

**Methods:**

We used a multistep process and multidisciplinary team of maternal-fetal medicine physicians, a behavioral economist, and researchers with expertise in behavioral interventions to design Healthy Beyond Pregnancy. We assessed the usability of the program with the Post-Study System Usability Questionnaire (PSSUQ), a validated 7-point scale, and semistructured interviews with postpartum women. We then conducted a feasibility trial to determine the proportion of eligible women who were willing to participate in an RCT of Healthy Beyond Pregnancy and the proportion of women willing to complete the Web-based program. Exploratory outcomes of the pilot trial included attendance at the postpartum visit, uptake of long-acting reversible contraception, and uptake of any contraception.

**Results:**

The median PSSUQ score for Healthy Beyond Pregnancy was 6.5 (interquartile range: 6.1-7) demonstrating high usability. Semistructured interviews (n=10) provided in-depth comments about users’ experience and further improved the program. A total of 34 postpartum women with Medicaid insurance were approached for the pilot trial, and 30 (88%) were consented and randomized. All women randomized to Healthy Beyond Pregnancy completed the Web-based program, had text-enabled cell phones, and were willing to receive text messages from the study team. Women in the Healthy Beyond Pregnancy arm were more likely to return for a postpartum visit compared with women in the control arm with 85% of women in Healthy Beyond Pregnancy returning versus 53% in the control arm (odds ratio in the Healthy Beyond Pregnancy group: 5.3; 95% CI 0.9-32.0; *P*=.06).

**Conclusions:**

We have developed a highly usable and acceptable Web-based program designed to increase attendance at the postpartum visit. Our pilot trial demonstrates that women are willing and able to participate in a randomized trial of a Web-based program and text messaging system.

**Trial Registration:**

Clinicaltrials.gov NCT03296774; https://clinicaltrials.gov/ct2/show/NCT03296774 (Archived by WebCite at http://www.webcitation.org/6tpgXFzyk)

## Introduction

During the postpartum visit, health care providers address a number of issues with both short- and long-term implications for maternal and child health. Clinicians counsel about contraceptive options, provide breastfeeding support, screen and refer for postpartum mood disorders, screen for cardiometabolic consequences of pregnancy complications, and discuss interconception care. They also connect women with primary care providers.

Attendance rates for the postpartum visit are markedly lower for women with limited economic resources [1]. In the United States, Medicaid provides health coverage to low-income adults, children, and pregnant women. Nationally, approximately, 50% to 60% of women with Medicaid insurance return for a postpartum visit, compared with over 80% of women with private insurance [2]. Medicaid programs serve pregnant women who are particularly vulnerable to poor health outcomes, and thus, this gap is critical.

The reasons for noncompliance with the postpartum visit are complex [1]. Women site a lack of transportation and childcare as contributing factors, as many clinics do not provide childcare during appointments. Women also indicate that they are unsure why the postpartum visit is important for their health [3-5]. This suggests that our care model does not engage all women to make good health care decisions postpartum and is disproportionately failing our most vulnerable mothers and infants. Innovative solutions that account for difficulty in making smart health decisions are imperative.

The field of behavioral economics acknowledges that people do not make exclusively rational choices. Immediate gratification, cognitive and psychological biases such as bounded rationality or status quo bias, and social norms profoundly influence decision making. Drawing on insights from psychology and decision science, the field of behavioral economics has examined how these biases can be modulated through carefully designed interventions [6]. Increasingly, these insights are influencing the health sciences as researchers seek more effective health interventions and health policy [7-14]. Given this, we developed an innovative Web-based tool, Healthy Beyond Pregnancy, with text messaging that incorporates empirically derived concepts of behavioral economics to improve adherence rates to the postpartum visit. We opted for a Web-based tool with text messaging, as low-income women between the age of 18 and 29 years use the Web-based application and send and receive text message more frequently than any other demographics [15]. The primary objectives of this pilot study were to (1) refine and assess the usability of Healthy Beyond Pregnancy and (2) assess the feasibility of a randomized controlled trial (RCT) of the intervention.

## Methods

### Phase 1: Development and Assessment of the Usability of Healthy Beyond Pregnancy

#### Theoretical Grounding and Description of Intervention

**Figure 1:**
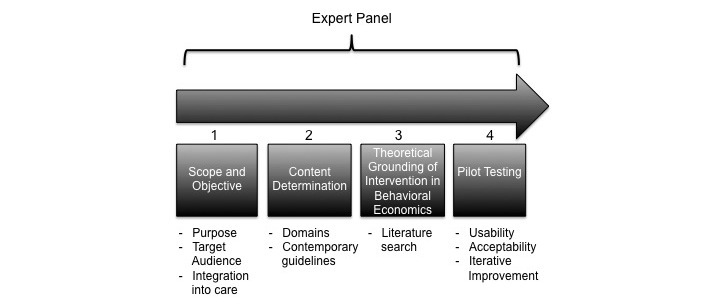
Conceptual steps used to develop and test Healthy Beyond Pregnancy.

We used a multistep process and multidisciplinary team of maternal-fetal medicine physicians, a behavioral economist, certified lactation consultant, and researchers with expertise in behavioral interventions to design Healthy Beyond Pregnancy. The broad conceptual steps that we used to develop and test Healthy Beyond Pregnancy are illustrated in Figure 1. The key behavioral economic concepts that informed Healthy Beyond Pregnancy and how they are implemented in the program include the following: (1) bounded rationality and information overload, (2) status quo bias or lack of self-control, (3) hovering or limited attention, and (4) framed incentives. These concepts are outlined below in detail.

First, bounded rationality and information overload indicates that patients’ decision making is hampered by the overwhelming amount of information available and the difficulty in focusing on all of the information relevant to their care. Furthermore, the perception of personal relevance of the information presented will affect how the information resonates with the patient and how motivating it is toward healthy behavior. Given this, Healthy Beyond Pregnancy allows participants to define much of the content of their postpartum education and acknowledges that only 2 to 3 issues can be meaningfully addressed for most patients. The first step on the Healthy Beyond Pregnancy Web platform is a survey that assesses the participants’ postpartum concerns from a scale of 1 to 5—with 1 representing not at all important to 5 representing very important. Women are presented with the following list of postpartum issues: (1) postpartum contraception, (2) breastfeeding support, (3) postpartum mood, (4) bowel and bladder function after delivery, (5) sexual activity after delivery, (6) optimizing interpregnancy health, and (7) follow-up after pregnancy complications such as gestational diabetes, hypertension, or spontaneous preterm delivery. Women’s answers on the scale of 1 to 5 are entered into an automated algorithm, and they watch 2 to 4 videos that reflect their self-identified needs—women watch the videos that they scored as most important (4 or 5 on the scale). Women with a pregnancy complicated by gestational diabetes, hypertension, or preterm birth will also view videos about the implications of these pregnancy complications. Given the health benefits of planned and timed pregnancies as well as breastfeeding, if a participant indicates that she is not interested in any of the postpartum domains, she will be shown videos on contraception and breastfeeding. Women are given the option to watch more videos if they want.

Second, status quo bias/or lack of self-control indicates that patients often make time-inconsistent choices—they may plan to go for a postpartum visit but put off making the appointment because the immediate costs (altering the status quo) loom larger than the delayed benefits of the visit [16]. Healthy Beyond Pregnancy makes scheduling and committing to a postpartum visit a default option. After defining their postpartum concerns and watching the educational videos, participants schedule a visit. This contrasts with our care model where women are asked to call and schedule their postpartum visit after they leave the hospital. After they schedule the visit, participants use a stylus to sign a commitment contract to attend this visit. Commitment devices restrict the choices of a future self and increase the probability of adhering to a future behavior [14].

Third, hovering or limited attention indicates that several tasks and choices compete for patients’ attention. Patients need reminders to keep an action on the top of their mental stack [17]. Thus, Healthy Beyond Pregnancy participants receive nudging text messages to keep the importance of their postpartum concerns in the forefront of their minds. Text messages were sent within 48 hours of their initial discharge from the hospital and within 72 hours for their scheduled postpartum visit. Examples of nudging text messages are included (see Multimedia Appendix 1). After participants schedule and commit to the postpartum visit, they are asked to share a short message service (SMS)–enabled phone number. We use this number to send motivational text messages, links to educational content, and relevant support services, as well as reminders about the date and time of their prescheduled and committed postpartum visit.

Fourth, the framed incentives emphasize on the fact that the uptake of behaviors can be influenced by salient incentives with proper framing [18]. Healthy Beyond Pregnancy rewards women who return for a postpartum visit with a cash incentive [19]. As part of the commitment contract, participants are informed that they will receive a US $40 cash incentive if they attend their postpartum visit. Cash incentives have been found to be more powerful than other incentives because they allow the recipient to apply it toward something that is personally important to them [19]. Thus, with framing the incentive as a positive reward instead of a deductible, they do not have to pay, which has the potential to increase behavior change without increasing the magnitude of the cost. The Web-based portion of the intervention is available at the URL at the end of the references.

#### Usability Testing

##### Design and Participants

We recruited a convenience sample of 15 women from the postpartum floors of Magee-Womens Hospital. Participants were recruited in three groups of 5 with iterative improvements made to the website after each group of 5 completed their assessments. All participants used the website and completed a usability measure. A subset of women also participated in a semistructured interview. Women were interviewed until thematic saturation was reached, which occurred after interviewing 10 women.

##### Usability Assessment and Semistructured Interview

After viewing the Healthy Beyond Pregnancy program, participants completed a printed questionnaire to assess the website’s usability. Usability is defined as the extent to which a product can be used to achieve its stated goals with effectiveness, efficiency, and the satisfaction of the user [20]. We administered the Post-Study System Usability Questionnaire (PSSUQ), a validated measure to assess user satisfaction with system usability [21]. The PSSUQ consists of 19 items that are rated on a 7-point scale, with low scores indicating strong disagreement with the statement. The questionnaire has three subscales that assess (1) system usefulness, (2) information quality, and (3) interface quality. One question regarding error messages to fix problems was omitted, as it was not applicable.

After viewing the Healthy Beyond Pregnancy site, 10 participants also completed a semistructured interview. In the interviews, participants were asked about the strengths and weaknesses of the program, as well as recommendations for improvement. Interviews were audiotaped and transcribed for analysis. No field notes were taken, and transcripts were not returned to the participants. Interview questions are included (see Multimedia Appendix 2).

#### Analyses

##### Quantitative Analyses

For the PSSUQ, medians and interquartile ranges (IQRs) were calculated. A nonparametric test of trend (nptrend) was performed to compare scores across the three groups of 5. Analyses were completed using STATA 13 (StataCorp LLC).

##### Qualitative

To create the initial coding scheme, two investigators independently performed coding of two interviews to identify themes. The coding scheme was collaboratively modified after application of initial codes to two additional interviews. All interviews were then thematically recoded with the final coding scheme. We continued interviews until thematic saturation was reached (n=10). Qualitative coding was organized using ATLAS.ti 4.2.

### Phase 2: Pilot Trial to Assess Feasibility of Randomized Controlled Trial of Healthy Beyond Pregnancy

#### Study Design

We conducted a pilot RCT to test the feasibility of randomizing postpartum women to Healthy Beyond Pregnancy or usual care. The trial was conducted at Magee-Womens Hospital, a large maternity hospital that provides care to women in Western Pennsylvania. The institutional review board of the University of Pittsburgh approved the trial (PRO16090292).

Inclusion criteria for the trial were (1) postpartum 6 to 72 hours from their delivery, (2) aged 18 to 50 years, (3) receipt of prenatal care through the Magee-Womens Hospital outpatient obstetrical clinic, and (4) “UPMC for You” Medicaid insurance. Women were excluded if they delivered in less than 24 weeks, experienced a fetal or neonatal death, did not speak English, or did not have a text-enabled phone. We recruited for the trial from November 2016 to February 2017.

Study investigators (KPH and FLF) who were part of the clinical team identified women eligible for the study. Participants were then approached for the study, consented, and randomized on the postpartum floor. We used computer-generated randomization to assign participants to Healthy Beyond Pregnancy or usual care in a 1:1 ratio. The Healthy Beyond Pregnancy program is described in detail in phase 1. It is not embedded in other parts of the hospital care and was administered by the members of the study team. The control arm received routine clinical care. This includes a reminder in their discharge paper work to call their clinic for a postpartum visit in 3 to 8 weeks.

#### Measures

##### Feasibility of Randomization

Our primary outcome included the proportion of eligible patients who consented to the study and the number of women randomized to Healthy Beyond Pregnancy who completed the Web-based program. We also assessed whether patients would recommend the program to a friend.

Exploratory outcomes included the proportion of women who attended a postpartum visit within 21 to 56 days after delivery and had an uptake of long-acting reversible contraception (LARC) and some form of contraception. The 21- to 56-day period is consistent with the Healthcare Effectiveness Data and Information Set (HEDIS) definition of a postpartum visit.

##### Demographic and Clinical Variables

Maternal and clinical data were abstracted from the medical records. These included maternal age, race, parity, gestational age at delivery, mode of delivery, opiate use during pregnancy, and number of prenatal visits.

#### Statistical Analysis

Outcomes were described using means or proportions. Demographic and clinical characteristics were compared between study arms using either independent sample *t* test or chi-square test. Differences in exploratory outcomes—attendance at the postpartum visit, receipt of LARC, or receipt of any birth control method other than condoms—between study arms were compared using univariate logistic regression. Importantly, this study was not powered to detect differences between groups in exploratory clinical outcomes such as adherence to the postpartum visit, and given the small sample size, multivariable modeling was not performed.

## Results

### Phase 1

A total of 15 women (three groups of 5 based on the timing of enrollment) participated in usability assessment of the Healthy Beyond Pregnancy website. Iterative improvements were made after each group of 5 completed their assessments. Participants were postpartum women aged between 22 and 38 years. Users spent between 9 to 15 min completing the program.

The median PSSUQ score was 6.5 (IQR: 6.1-7) demonstrating high usability. Each of the subscales also demonstrated high usability scores—median score on system quality was 6.6 (IQR: 6.25-7), median score on information quality was 6.6 (IQR: 5.8-7), and the median score on interface quality was 7 (IQR: 6-7). Although the median overall PSSUQ score improved over the course of the three usability testing groups—Group 1: 6.5 (6.5-7), Group 2: 6.7 (6.5-7), and Group 3: 6.8 (6.5-7)—this was not significant (*P*=.52).

The semistructured interviews (n=10) provided more in-depth comments about users’ experience of using Healthy Beyond Pregnancy. The interview questions are provided in Multimedia Appendix 2).

The median interview time was 14 min (IQR: 10-15). Overall comments were positive with 90% (n=9) of women indicating that they would recommend the program to a friend who had just delivered. Important design improvements also emerged from these interviews. For example, we restructured our scheduling calendar to make available appointments easier to identify, changed the language in the tablet computer to make it more accessible (preterm birth became delivery in less than 37 weeks), and added a written summary of the personalized information provided by Healthy Beyond Pregnancy. Table 1 highlights themes identified by at least 40% of participants that emerged on the benefits of Healthy Beyond Pregnancy from the semistructured interviews.

**Table 1:** Participants’ perspectives on utility of Healthy Beyond Pregnancy.

Theme	Participants identifying theme (n=10), n (%)	Examples
Help personalize postpartum care	4 (40)	“Provided enough information to help me focus my thoughts for postpartum visit.” (ID: HBP7)
		“Helps me focus on the problems that are relevant to me.” (ID: HBP9)
Decrease the stress of postpartum period	6 (60)	“It is really nice to schedule your appointment.” (ID: HBP2)
		“Great to not have the stress of calling for an appointment.” (ID: HBP4)
Program is easy to use	9 (90)	“I love that I can breastfeed while using the website.” (ID: HBP3)
		“You don’t have to struggle to get through the program.” (ID: HBP6)
Highlights issues you are not focusing on	8 (80)	“There is so much on your mind...this reminds you about important issues.” (ID: HBP1)
		“This reminded me not to forget about important issues for my health.” (ID: HBP10)

### Phase 2

#### Primary Outcomes

**Figure 2:**
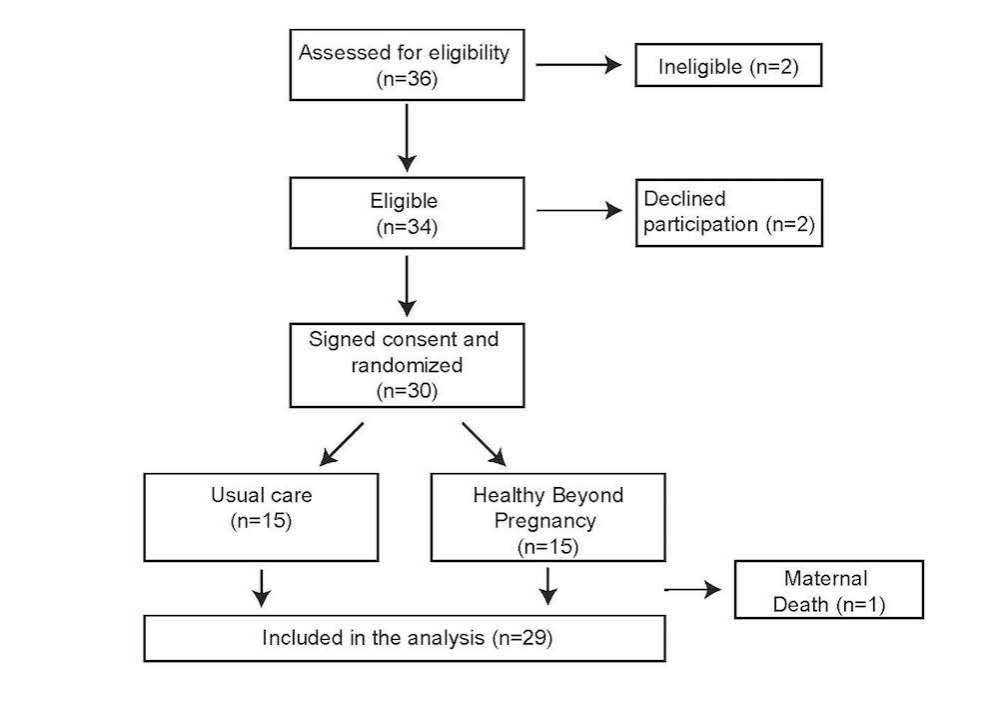
Participant flow in Healthy Beyond Pregnancy feasibility study.

A total of 34 women were approached for the pilot trial, and 30 (88%) were consented and randomized (Figure 2). Importantly, all women randomized to Healthy Beyond Pregnancy were willing to complete the entire Web-based program, had text-enabled cell phones, and were willing to receive text messages from the study team. All participants randomized to Healthy Beyond Pregnancy indicated that they would recommend the program to a friend who had just delivered. The baseline demographics and clinical characteristics are outlined in Table 2.

**Table 2:** Demographic and clinical characteristics at randomization.

Variables		Usual care (n=15)	Healthy Beyond Pregnancy (n=15)	*P* value
Maternal age in years, mean (SD^a^)		29.6 (4.3)	27.9 (5.0)	.31
**Race, n (%)**				
	African American	6 (40)	10 (67)	.26
	White	8 (53)	5 (33)	
	Asian	1 (7)	0 (0)	
Nulliparous, n (%)		1 (7)	6 (40)	.03
Gestational age in weeks, mean (SD)		38.5 (0.4)	37.9 (0.5)	.30
Vaginal delivery, n (%)		7 (47)	11 (73)	.14
Number of prenatal visits, mean (SD)		8.5 (0.9)	10.4 (0.6)	.08
Opiate use, n (%)		7 (47)	2 (13)	.11

^a^SD: standard deviation.

#### Exploratory Outcomes

All participants enrolled in the trial had UPMC Medicaid insurance, and thus, we were able to ascertain our clinical outcomes on all patients, as they must seek care through a UPMC facility, all of which use a common electronic medical record system. There was a trend toward more women in the Healthy Beyond Pregnancy arm returning for a postpartum visit compared with women in the control arm, that is, 85% of women in Healthy Beyond Pregnancy returning versus 53% in the control arm—odds ratio of 5.3 (95% CI 0.9-32.0; *P*=.06). Importantly these results were not significant. All exploratory outcomes are outlined in Table 3.

**Table 3:** Exploratory outcomes.

Outcomes	Usual care (n=15), n (%)	Healthy Beyond Pregnancy (n=14), n (%)	Odds ratio (95% CI)	*P* value
Attended postpartum visits	8 (53)	12 (85)	5.3 (0.9-32.0)	.06
LARC^a^	4 (26)	3 (21)	0.8 (0.2-4.2)	.74
Any contraception	11 (73)	11 (78)	1.3 (0.3-7.7)	.73

^a^LARC: Long-acting reversible contraception.

## Discussion

### Principal Findings

We have developed a highly usable and acceptable Web-based program designed to increase attendance at the postpartum visit. Our pilot trial demonstrates that women are willing and able to participate in a randomized trial of a Web-based program and text messaging system. Furthermore, we saw a trend toward increased compliance with the postpartum visit among women in the Healthy Beyond Pregnancy arm. These results were not significant.

Despite observing a trend toward increased postpartum visit compliance, we found similar rates in our two study arms in these contraception outcomes. There are several possible reasons for this. Some trial participants had postpartum tubal ligations before randomization—this included 26% (n=4) and 7% (n=1) of our control group and Healthy Beyond Pregnancy group, respectively. As there are benefits beyond contraception to the postpartum visit, we opted not to exclude these women from the trial. Furthermore, at our institution, some women with UPMC for You insurance qualify for an etonogestrel implant (a LARC method) before discharge. This, however, is not true for other Medicaid insurance products. Finally, women can also opt to get a single medroxyprogesterone injection or a prescription for 3- to 6-month supply of combined oral contraceptive pills before discharge from the hospital. In addition, 20% (n=3) of women in our control arm fall into this category. Although there is documentation of contraception provision for these women, without establishing postpartum care, these women are at risk of not being able to establish a long-term contraception plan. Further investigation of our study tool with a larger sample size and a longer follow-up period is needed to help understand the impact of Healthy Beyond Pregnancy on contraception use after delivery.

The willingness of Medicaid recipients to participate in a trial designed to improve compliance with the postpartum visit is important because attendance rates for the postpartum visit are lower for women with limited resources, potentially contributing to health disparities. Minority women and women of lower socioeconomic status are at significantly increased risk of unintended pregnancies, short interpregnancy interval, and short duration of breastfeeding. The maternal and child health outcomes related to unintended pregnancies, short interpregnancy interval, and short duration of breastfeeding are well documented, and importantly, these measures can be impacted during the postpartum period [22-31]. Furthermore, Internet and mobile phone–based interventions may be particularly successful with our target population, as low-income and non-Hispanic black women aged between 18 and 29 years send and receive text messages more frequently than any other demographics [15].

An important component of our intervention is that it is designed to be both affordable and scalable. There are a number of other postnatal interventions, including patient education booklets, home visits, prescheduling visits, and cash incentives, that have been designed to improve postpartum care in the developed world [3,9,32-35]. Only two of these studies used an RCT study design, limiting conclusions about effectiveness. These data suggest home visits are effective in improving compliance with postpartum visits. Home visits, however, are expensive and difficult to scale. Patients can complete the Healthy Beyond Pregnancy program independently, and the text messaging system can be automated. Additionally, other investigators have used Internet-based and text messaging interventions in the postpartum period successfully [36-38]. Finally, incentives are feasible, as many health plans already offer lower deductibles when preventative care milestones are met.

### Limitations

It is important to emphasize that this was a usability study and pilot RCT to assess the feasibility of enrolling patients in a large study of Healthy Beyond Pregnancy. We enrolled patients immediately postpartum. This is a busy and potentially emotionally charged time for women. Furthermore, our intervention targets women from disadvantaged socioeconomic backgrounds who have additional stressors in the immediate postpartum period. Before pursuing a large RCT, we wanted to assess our ability to consent, randomize, and retain women in our study. Given the pilot nature of the project and small sample size, our findings regarding adherence to the postpartum visit must be viewed with caution. It is also important to note that our Healthy Beyond Pregnancy arm had significantly more nulliparous women than our control arm. Women with multiple children may be less likely to attend a postpartum visit. Thus, the greater proportion of nulliparous women in the Healthy Beyond Pregnancy arm may contribute to our increased adherence to the postpartum visit in this group. Nevertheless, the information garnered from this pilot trial will be important for a future efficacy trial. A larger trial will allow us to look definitively at attendance at the postpartum visit, as well as important health outcomes such as breastfeeding duration and provisions of LARC. These outcomes are critical to improve a number of short- and long-term maternal and child health outcomes.

### Conclusions

We have developed a usable and acceptable Web-based program designed to increase attendance at the postpartum visit. Our pilot trial demonstrates that women are willing and able to participate in a randomized trial of a Web-based program and text messaging system. Importantly, although our trial was not powered to detect difference in attendance at the postpartum visit, we observed a trend toward increased compliance with the postpartum visit among women randomized to Healthy Beyond Pregnancy. A large RCT is needed to determine whether attendance can be increased robustly and whether this would translate into improved health outcomes.

## Appendix

Multimedia Appendix 1 Sample nudging text messages.

Multimedia Appendix 2 Semistructured interview questions.

Multimedia Appendix 3 CONSORT-eHEALTH V1.6.

## Abbreviations

IQR: interquartile range
LARC: long-acting reversible contraception
PSSUQ: Post-Study System Usability Questionnaire
RCT: randomized controlled trial
