# Supplementary material for: Healthy Beyond Pregnancy, a Web-Based Intervention to Improve Adherence to Postpartum Care: Randomized Controlled Feasibility Trial
Source: JMIR Hum Factors. 2017 Oct 10;4(4):e26. doi: 10.2196/humanfactors.7964 (PMC5654734; doi:10.2196/humanfactors.7964)
Supplement: Multimedia Appendix 1 [file humanfactors_v4i4e26_app1.pdf]

Postpartum Concern	Sample Text Messages
Birth Control	• Planned pregnancies are healthy ones. Space pregnancies one year apart.<br>• Intrauterine devices (IUD) and birth control implants work well and last for 3-10 years. They are easy to use and can be removed at anytime.<br>• 7/10 women like you are still using an IUD two years after getting one!
Breastfeeding Support	• Congratulations on your decision to breastfeed! If you are having trouble reach out to our breastfeeding experts: (412) XXX-XXXX<br>• 4 out of 5 women breastfeed after leaving the hospital- you can do it!<br>• Breast milk helps your baby fight off infection and lowers your baby's risk of having asthma or allergies. Give your baby the best start!<br>• Breastfeeding is not just for baby. It decreases your risk of diabetes, heart disease, and help women lose weight postpartum.
Postpartum Mood	• Most new moms feel mood swings and can be weepy during the first 2-3 weeks after delivery. If it doesn't get better or gets worse call your doctor.<br>• Are you feeling angry or sad? Do you lack interest in your baby or feel hopeless? These can be signs of postpartum depression.<br>• About 1 in 10 women get postpartum depression—if you have concerns please call your doctor.

Outcome	Usual Care<br>(n=15)	Healthy Beyond<br>Pregnancy<br>(n=14)	OR (95% CI)	p value
**Attended Postpartum Visit**				
	8 (53.3)	12 (85.7)	5.3 (0.9, 32.0)	0.06
**LARC**				
	4 (26.6)	3 (21.4)	0.8 (0.2, 4.2)	0.74
**Any Contraception**				
	11 (73.3)	11 (78.5)	1.3 (0.3, 7.7)	0.73
