# Supplementary material for: Healthy Beyond Pregnancy, a Web-Based Intervention to Improve Adherence to Postpartum Care: Randomized Controlled Feasibility Trial
Source: JMIR Hum Factors. 2017 Oct 10;4(4):e26. doi: 10.2196/humanfactors.7964 (PMC5654734; doi:10.2196/humanfactors.7964)
Supplement: Multimedia Appendix 2 [file humanfactors_v4i4e26_app2.pdf]

Semi-structured interview

Now that you have used the Healthy Beyond Pregnancy, we are interested in learning what you like and disliked about the web site.

1. Can you tell me what, if anything, you liked about the website?
2. Can you tell me what you liked least about the website?
3. Could you navigate through the site?
4. Do you like the way the website looks?
5. What did you think about the information on the website?
6. What did you think about the videos on the website?
7. Do you think the information you got from this website is accurate?
8. What would you change about the website?
9. Would you recommend this program to a friend who had just delivered?
10. Do you have any other comments?
